# *Cryptosporidium* spp. and *Giardia* spp. in Free-Ranging Introduced Monk Parakeets from Santiago, Chile

**DOI:** 10.3390/ani11030801

**Published:** 2021-03-12

**Authors:** Alejandra Sandoval-Rodríguez, Daniela Marcone, Raúl Alegría-Morán, Matilde Larraechea, Karina Yévenes, Fernando Fredes, Cristóbal Briceño

**Affiliations:** 1 Programa de Doctorado en Ciencias Silvoagropecuarias y Veterinarias, Campus Sur Universidad de Chile, Santa Rosa 11315, La Pintana, Santiago CP 8820808, Chile; alesandovalrodriguez@gmail.com; 2 Animal Preventive Medicine Department, Faculty of Animal and Veterinary Sciences, University of Chile, Santiago CP 8820808, Chile; dmarconed@gmail.com (D.M.); or ralegria@profesores.upv.cl (R.A.-M.); matilde.larraechea@gmail.com (M.L.); kariyevenescoa@gmail.com (K.Y.); ffredes@uchile.cl (F.F.); 3 Faculty of Agricultural and Environmental Sciences, Pedro de Valdivia University, Santiago CP 8370007, Chile

**Keywords:** Cryptosporidium, Giardia, invasive species, Monk Parakeet, *Myiopsitta monachus*, parasites, protozoa, synanthropic species, zoonoses

## Abstract

**Simple Summary:**

Monk Parakeets are medium-sized parrots that were internationally traded as pets and that as a byproduct have become invasive species in 19 countries. This is the case of Chile, where Monk Parakeets have thrived in the city of Santiago. *Cryptosporidium* spp. and *Giardia* spp. are parasites that can affect the digestive system of a wide variety of animals, including humans and birds. This study sought to determine the occurrence of these parasites within Monk Parakeets from the city of Santiago. To do this, 207 fecal samples from Monk Parakeet nestlings that were captured during the summer seasons of 2017 and 2018 were analyzed. Environmental data related to the trees in which the nestlings were captured was studied in order to the determine the existence of areas more prone to have infected parakeets and whether certain environmental variables influence the presence or absence of these parasites in Monk Parakeets. In total, 33 samples were positive to the presence of one or both parasites. Of the 33, 10 nestlings (30%) were infected with *Cryptosporidium* spp. and 25 (76%) with *Giardia* spp. Two nestlings were infected with both parasites (6%). Environmental analyses revealed that pruned trees might constitute a protective factor against infection with these parasites. These findings emphasize Monk Parakeet’s potential role as a disease disseminator, especially in urban environments.

**Abstract:**

Monk Parakeets (*Myiopsitta monachus*) are medium-sized parrots that due to international pet trade currently exist as invasive species in 19 countries globally. Such is the case of Chile, where Monk Parakeets have thrived in the city of Santiago. *Cryptosporidium* spp. and *Giardia* spp. are worldwide distributed gastrointestinal parasites whose potential hosts include birds and humans. The present study sought to determine the presence of these pathogens in Monk Parakeets from Santiago. During the austral summers of 2017 and 2018, 207 Monk Parakeet nestlings were captured, and fecal samples were studied via microscopical analyses. Environmental data related to the trees in which the nestlings were captured were analyzed to establish the existence of infection clusters. Associations between spatial clusters, environmental variables, and the presence or absence of these pathogens were explored. In total, 33 samples were positive to the presence of one or both protozoa. Of the 33, *Cryptosporidium* spp. oocysts were detected in 10 nestlings (30%) while *Giardia* spp. cysts were detected in 25 (76%). Two nestlings presented poly-parasitism (6%). Statistical analyses established pruned trees as a potential protective factor against infection with these parasites. The present study corresponds to the second report of *Cryptosporidium* spp. in Monk Parakeets in Chile and the first worldwide report of *Giardia* spp. in these birds, emphasizing Monk Parakeet’s potential role as a reservoir and pathogen disseminator, especially in urban environments.

## 1. Introduction

Biological invasions are recognized as a major cause of biodiversity loss and one of the main generators of ecological novelty [1,2]. Invasive species have been linked to the emergence of diseases in native populations and recently have been acknowledged as relevant factors that contribute to the spread of zoonoses [3,4,5].

Monk Parakeets (*Myiopsitta monachus*) are gregarious, medium-sized parrots naturally distributed in Paraguay, Uruguay, Bolivia, southern Brazil, and northern and central Argentina. However, due to the international pet trade, this bird can nowadays be found as an invasive species in 19 countries of Africa, Asia, Europe, and America [6,7,8,9]. Monk Parakeets’ invasive success can be associated with the unique behavioral and ecological characteristics of the species [10,11]. Their great reproductive capacity and ability to prosper in novel environments can be attributed to, among other things, their reproductive, nesting, and dietary habits [6,7,10,11,12,13,14,15].

Several negative impacts are associated with the presence of Monk Parakeets, both in countries where they are considered a native species and in countries they have invaded [12,14]. These impacts are often associated with economic losses derived from Monk Parakeet’s activity [16]. Parakeets sometimes utilize human structures such as electric posts to build their nests, which can lead to power outages [12]. Their generalist diet often results in considerable economical costs for agricultural fields. For instance, in Argentina, these birds cause losses of up to 1 billion dollars every year due to crop damage [17]. Studies on Monk Parakeets have, so far, focused mainly on its economic impact or ecological traits, being that reports on this invasive bird’s sanitary state are scarce. It is only recently that the presence of pathogens such as the beak and feather disease virus and the parasite Leucocytozoon were described in Monk Parakeets from Southern Spain [18,19].

Monk Parakeets were introduced in Santiago of Chile in the early 1970s, when parakeets were released in the northeast area of Santiago, from where they started to spread to the rest of the territory [20]. Nowadays, Monk Parakeets are considered to be one of the most harmful species of invasive birds in Chile due to their negative impact associated with agricultural, fruit, and ornamental trees [5]. Despite this, scarce research has been conducted to establish the real impact of Monk Parakeets as an invasive species in Chile [5].

Recently, the presence of *Cryptosporidium* spp. in fecal samples from Monk Parakeets captured in Northeast Santiago, Chile, was reported [5]. A second report described interactions between Monk Parakeets and other bird species in Santiago, Chile, revealing that parakeets and other species of birds can coexist pacifically [20]. Interestingly, this study showed that, as observed in other countries [21], other bird species make use of Monk Parakeet nests for breeding, evidencing Monk Parakeet’s role as an ecosystem engineer [20].

*Cryptosporidium* spp. often coexists with *Giardia* spp., another gastrointestinal parasite. Both are transmitted via orofecal route by ingestion of water or food contaminated with (oo)cysts [22,23,24]. Although infections with these parasites can be asymptomatic or produce imperceptible clinical signs, immunocompromised individuals can eventually become sick and exhibit symptoms such as diarrhea, abdominal pain, nausea, vomiting, and fever [25,26]. In psittacine birds, more severe cases of cryptosporidiosis can be associated with beak and feather disease, while giardiasis may lead to weight loss, feather picking, and overall failure to thrive [27]. Both parasites can be detected and isolated in the environment, contaminated water sources being one of the main sources of contagion [22,23,24]. *Cryptosporidium* spp. and *Giardia* spp. have also been detected in soil samples from public parks in Madrid, Spain [28]. Cryptosporidiosis and giardiasis are considered neglected diseases by the World Health Organization [29]. This is of importance since *Cryptosporidium* spp. and *Giardia* spp. infections primarily occur in developing countries where infection outcomes can be influenced by factors such as poverty and lack of access to appropriate resources. Disease by these pathogens can impede the proper physical and socioeconomic development of those affected by them [29]. It is also an important issue to be considered from a health perspective, due to the interdependence of human, animal, and environmental health [30,31].

Considering that biological invasions can be a source of pathogens, some of them zoonotic, and can thus eventually affect the health of people [3,32,33,34], and that Monk Parakeets are synanthropic birds, keeping a close contact with human populations by building their nests within urban public squares and parks [20,21], investigating the sanitary state of these invasive birds becomes important. Therefore, the aim of this study was to perform a thorough inquest into the occurrence of these pathogens within Monk Parakeets located in Santiago. We additionally sought to determine the existence of infection clusters within the city and to study the correlation between environmental variables associated with the trees in which Monk Parakeets nest and positive rates of infection.

## 2. Materials and Methods

### 2.1. Study Area

The sampling area compromised twenty-one municipalities of Santiago (33°27′ S; 70°38′ W), the capital city of Chile. Santiago is located in the Metropolitan Region in Central Chile, a Mediterranean bioclimatic zone defined by dry summers, wet winters, and interannual variability caused by the El Niño-Southern Oscillation phenomenon [35]. Mean annual temperature is 13.2 °C, and mean annual precipitation is 531 mm [36]. Vegetation occurs in an assortment of *Acacia caven* shrubland on lower hillslopes and evergreen sclerophyllous forest on watersheds and south-facing slopes [36,37]. The Metropolitan Region is currently divided into 52 municipalities and is the highest densely populated region in the country, concentrating 40% of the national population with 7,112,808 inhabitants and a density of 462 people/km^2^ [38]. Due to its accelerated and unorganized expansion, Santiago is characterized by urban sprawl and deep environmental degradation [39]. Green areas within the city are highly stratified, from a total of 3825 ha, 62% (2387 ha) are distributed in only 3% of the total green areas. These areas are associated with large public parks [40].

### 2.2. Study Species

Monk Parakeets (*Myiopsitta monachus*) are medium-sized, sexually monomorphic parrots originally distributed in the southeastern area of South America, specifically in Paraguay, Uruguay, Bolivia, southern Brazil, and northern and central Argentina [6,41]. As a consequence of the international pet trade, Monk Parakeets have been introduced into several countries, becoming an invasive species in 19 countries of Africa, Asia, Europe, and America, including Chile [6,7,8,9]. The invasive success this psittacine bird has exhibited can be attributed to several behavioral and ecological traits. More notably, Monk Parakeets are the only species among the order Psittaciformes that does not rely on preexisting cavities to nest, since they are able to build their own communal nests [7,12,42]. Furthermore, they feed on a flexible diet, allowing them to exploit a wide variety of fruits and seeds [13]. Additionally, their gregarious behavior leads them to engage in communal parenting, increasing their possibilities to avoid predatory attacks [6,7,12]. Ultimately, they display great tolerance to human presence and urban perturbations, often building their nests in public squares and parks [7,10,11]. All these traits provide a great capacity to adapt to different environments and lead to an accelerated population growth [11,14]. Further, their reproductive success seems to be higher in introduced versus native distributions [15].

### 2.3. Nestling Sampling and Processing

**Figure 1:**
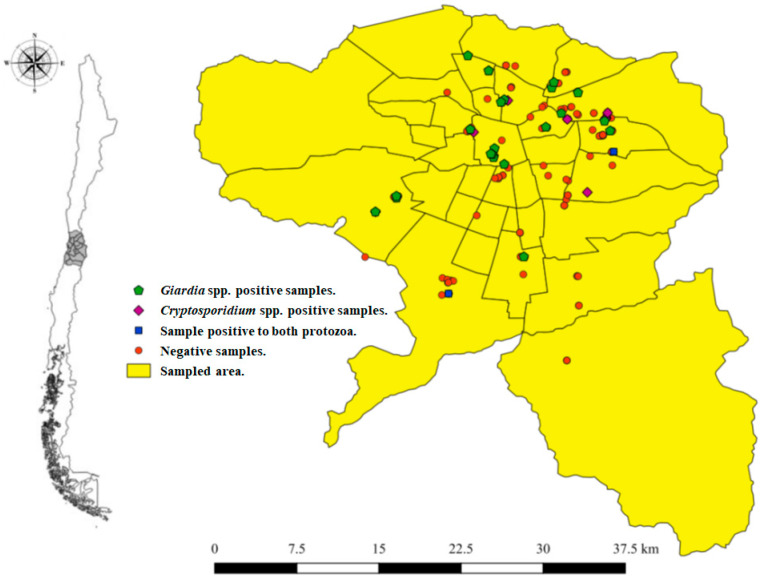
Sampled area within the city of Santiago, Chile. Green pentagons represent *Giardia* spp. positive samples; purple rhombus represents *Cryptosporidium* spp. positive samples; blue squares represent samples that were positive to both parasites; red dots represent negative samples.

Sampling took place during the austral Monk Parakeet’s breeding season: November and December of 2017 and 2018. Twenty-one municipalities of Santiago, Chile, were opportunistically sampled for the study (Figure 1). Monk Parakeet nestlings were considered as the sample unit and were assumed to represent the nest inhabitants’ sanitary condition. Nestlings were manually counted and captured from their nest and accessed through a hydraulic aerial platform. Only one individual was captured per nest and per tree.

Since parakeet sampling depended upon the number of trees with occupied nests per municipality, accessibility to nests, technical support provided by each municipality, and daily possibilities to sample, among others, the sampling process was of convenience.

Nestlings were sacrificed via cervical dislocation following bioethical protocols. During the necropsy, individuals were aged and the distal portion of the digestive tract and its content was sectioned and preserved in 70% ethanol at 4 °C until further analyses. All procedures were conducted in compliance with national regulations established by Servicio Agrícola y Ganadero (SAG, Chilean Fish and Wildlife Service), under permit No. 716/2016, and bioethical and biosafety protocols issued by the Faculty of Animal Veterinary Sciences, University of Chile (Bioethical approval No. 19-2016 and Biosafety approval No. 82).

### 2.4. Protozoa Detection

Fecal content was extracted from the preserved digestive tract for macroscopic and microscopic parasitological analyses. For detection of *Cryptosporidium* spp. Oocysts, the modified Ziehl Neelsen method was employed using 500 µL of sediment extended onto a 1 cm × 0.5 cm slide [43]. *Giardia* spp. cysts were detected by means of modified Telemann method using Lugol’s stain and 100 µL of sediment extended onto a 1 cm × 0.5 cm slide [44].

### 2.5. Environmental Variables

For each nestling sample, the following environmental data related to the tree in which it was captured were recorded: municipality in which it was located, tree species, sanitary state of the tree (healthy/not healthy), tree management (pruned/not pruned), tree height, tree canopy diameter, diameter at breast height (DBH), number of nests per tree, nest size, nest height, number of chambers per nest, number of nestlings per nest, and GPS coordinates. Tree canopy diameter and DBH were measured using a 30 m measuring tape, while tree height and nest height were measured using a hypsometer. Health condition of trees was determined with the collaboration of academics at the Faculty of Forestry Sciences at the University of Chile.

### 2.6. Statistical Analyses

Descriptive statistics were performed to summarize each recorded variable. Considering the nature of the collected data (positivity to *Cryptosporidium* spp. oocysts and positivity to *Giardia* spp. cysts), three logistic regression model analyses were performed, one for each pathogen and one for the combined two protozoan parasites (model including the results of both pathogens) [45]. Univariate logistic regression analyses were performed to assess the relationship between all recorded variables and the positivity to the pathogens (for each model). Spearman correlation, Pearson chi-square, and Fisher’s exact test were performed to assess for collinearity and potential confounding factors, setting significance on *p* < 0.05. Variables with a *p*-value ≤ 0.25 were included in a multivariable logistic regression model (liberal criteria). A stepwise backward elimination procedure was conducted, using the log likelihood ratio test (LRT); the model with the lowest LRT was selected as the final model (in the three built models). Likewise, variables whose regression coefficients were not significant (*p* > 0.05) were removed from the multivariable logistic regression [46]. Non-significant variables, which produced a change greater than 20% in the regression coefficients of the significant variables when removed, were retained in the model to adjust for confounding factors. All the biologically feasible interaction variables were included in the model design [45]. The convergence of the models was set at a value of epsilon = e^−16^ in order to increase restrictions on models and to secure the representativity and power of results. Goodness-of-fit of the final model was evaluated using the Hosmer and Lemeshow test [47].

Biologically logical interactions between variables that fulfilled the liberal criteria were also analyzed. All categorical variables were analyzed using the dummy variables approach [48]. Local clustering of positive samples was assessed by means of the Bernoulli model of the spatial scan statistic, considering a purely spatial cluster analysis [49,50]. Analyses were conducted using the RStudio and the statistical software R 3.3.1 [51] plus “lme4”, “ggplot2”, and “gcookbook” packages, and SatScan software version 9.4.2. [52]. Odds ratios, 95% confidence intervals, and *p*-values were computed.

Finally, statistically significant differences between sampling years was determined by calculating the prevalence difference, equivalent to an attributable risk or excess risk, and 95% confidence intervals, based on approximation and null hypothesis testing (prevalence difference equals to 0) [53]. This analysis was performed using R version 3.6.1 [51] and “fmsb” package [54].

## 3. Results

### 3.1. Protozoa Detection 

**Figure 2:**
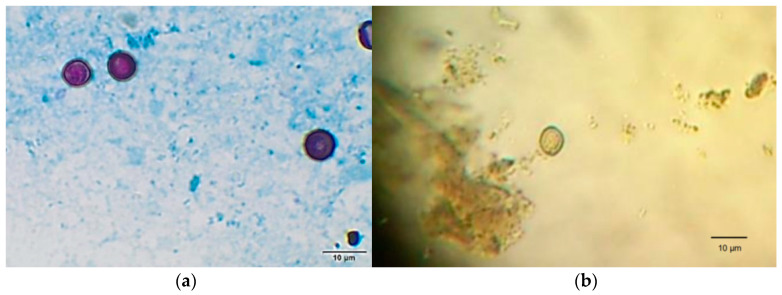
Microscopical analyses of intestinal content of Monk Parakeets. (**a**): *Cryptosporidium* spp. oocysts detected via Ziehl–Neelsen staining (1000×); (**b**): *Giardia* spp. cyst detected via Telemann method and stained using an iodine solution (400×).

A total of 207 Monk Parakeet nestlings were captured during the sampling period, 98 in 2017 and 109 in 2018, and a subsequent total of 207 stool samples were analyzed. Thirty-three samples (17%) were positive for the presence of protozoa. Total number of positive samples per parasite and per municipality can be seen in Table 1. Among the positive samples, 30% (10/33) presented *Cryptosporidium* spp. oocysts and 76% (25/33) presented *Giardia* spp. cysts (Figure 2). Poly-parasitism were detected in 2 out of the 33 positive samples (6%). Positivity to *Cryptosporidium* spp. was of 8.2% in 2017 (8/98) and 1.8% in 2018 (2/109). Positivity to *Giardia* spp. was 10.2% in 2017 (10/98) and 13.8% in 2018 (15/109). Overall, individuals that were positive to the presence of *Cryptosporidium* spp. oocysts had a regular load of 5 to 10 oocysts per 500 µL of analyzed sample, while those positive to *Giardia* spp. presented 1 to 5 cysts detected by microscopy per 100 µL of analyzed sample.

The presence of other parasites such as helminths and other protozoa was discarded via microscopical observation during the modified Telemann exam.

### 3.2. Environmental Variable Analyses

Sample records for each nestling were analyzed using the three mentioned logistic regression models. The *Cryptosporidium* spp. model (Table 2) shows borderline significance (*p* = 0.06) of pruned trees as a protective factor (odds ratio (OR) = 0.14, 95% CI lower limit (LL) = 0.01 and upper limit (UL) = 0.83), indicating a potential trend into the exploration of the other two models. Models for *Giardia* spp. show consistency of a significant role of pruning trees as a protective factor (OR = 0.25, 95% CI LL = 0.09 and UL = 0.71; *p* = 0.01). Nest height also showed a borderline significance (*p* = 0.07), but the OR and 95% CI show a non-causal association (Table 3). Finally, the number of nestlings per nest exhibited a significant positive association with *Giardia* spp. infection (OR = 1.59, 95% CI LL = 1.17 and UL = 2.17; *p* = 0.003). The model built for protozoans also shows a statistically significant association (*p* = 0.004) for pruning trees, with a protective role (OR = 0.26, 95% CI LL = 0.096 and UL = 0.62) (Table 4). Spearman correlation, chi-square, and Fisher’s exact test results show no collinearity and potential confounding factors among the variables included in the multivariable logistic regression models (*p* > 0.05).

### 3.3. Spatial Analyses

All 10 *Cryptosporidium* spp. positive samples, 25 *Giardia* spp. positive samples, and 33 Protozoan positive samples were distributed throughout the study area. The Bernoulli model of the spatial scan statistic detected several clusters, none of them significant. Only one of them, a cluster for *Giardia* spp., presented borderline significance (*p* = 0.06). This cluster included 9 sample points in a radius of 1.26 km, with a relative risk of 6.95, indicating a high risk of positivity at this site, located at Santiago, San Miguel, and San Joaquin municipalities.

### 3.4. Sampling Season Analysis

Prevalence values showed statistically significant differences (*p* < 0.05) for *Cryptosporidium* spp. in regard to sampling years, with higher prevalence in the sampling season of 2017 in comparison to 2018 (Table 5).

## 4. Discussion

Even though introduced Monk Marakeets have been inhabiting Chile for almost 50 years, not much is known about the local impact of this invasive bird [5,20]. The present study detected two gastrointestinal protozoa in Monk Parakeets from the city of Santiago and identified a possible protective factor associated with the absence of infection with these parasites. The findings of this work coincide with *Cryptosporidium* spp. oocysts found in adult Monk Parakeets from Lo Barnechea municipality, where 19.1% of sampled adult specimens were positive to the presence of the parasite [5]. The current findings, however, show that infection by *Cryptosporidium* spp. is not restricted to the municipality of Lo Barnechea or adult birds but can also occur in nestlings from different municipalities of Santiago. In addition to the detection of *Cryptosporidium* spp., the present study also reports the presence of *Giardia* spp. in intestinal samples of Monk Parakeets from the city of Santiago, corresponding to the first report of this parasite in this psittacine bird worldwide.

Monk Parakeet nestlings rely completely on their parents for feeding and do not leave the nest until they reach approximately 40 days of age [55]. Considering that *Cryptosporidium* spp. and *Giardia* spp. are orofecally transmitted parasites [23], that Monk Parakeets are gregarious animals that live in communal nests where they maintain close contact and practice collaborative parenting, and that they defecate within these nests [6,7,12], it is highly likely that nestlings acquired these pathogens by being in close contact with infected adult individuals or through their feces.

Detection of *Cryptosporidium* spp. and *Giardia* spp. in Monk Parakeet’s fecal samples is of relevance, as these widely distributed gastrointestinal parasites possess great zoonotic potential, exhibiting a wide range and variety of potential hosts that include mammals, fish, amphibians, reptiles, and birds [23,56,57,58,59,60,61,62]. Potential host species for these parasites often coincide, creating the possibility of coinfection events [22,24]. Such an event was detected in this study, as two of the sampled parakeets were infected with both *Cryptosporidium* spp. and *Giardia* spp. parasites.

When studying the association between the presence of these pathogens and environmental variables recorded at Monk Parakeet’s nesting sites, it was possible to observe a negative association between parasite detection and pruned trees. This could indicate that pruned managed trees might constitute a protective factor against *Cryptosporidium* spp. and *Giardia* spp. infections. Considering that pruning is of great importance when it comes to maintenance of tree health [63], it is possible that this activity helps creating a less suitable environment for the survival of (oo)cysts in parakeet nests and surroundings, and therefore reducing the risk of infection. One way pruning trees could be contributing to lower infection rates is by allowing for higher U.V. light penetration toward nests. Solar U.V. light has been shown to have the power to inactivate *Cryptosporidium parvum* oocysts [64,65] and could thus be contributing to reduce the infective (oo)cysts load within nests. Another factor that could potentially be influencing this finding is pruning season. If trees are pruned during the winter season and some nests are destroyed during the process, it could be possible that some of the nests sampled during Monk Parakeet’s breeding season (spring and summer) corresponded to recently built structures. Due their novelty, new nests could present lower loads of accumulated (oo)cyst, which could translate into lower risk of infection.

Interestingly, a statistically significant positive association between the number of nestlings per nest and presence of *Giardia* spp. was observed. This association, however, was not detected in the case of *Cryptosporidium* spp., nor when both protozoa were analyzed together. A similar situation occurred in the case of sampling season and infection rates. It was possible to detect a statistically significant difference between the amount of *Cryptosporidium* spp. positive samples collected during the 2017 sampling season and the 2018 sampling season. This difference, however, was not detected in the case of positive samples to *Giardia* spp. Further investigation is warranted to determine if these findings are associated with specific environmental variables or are casual results, a consequence of the sampling limitations of the present study.

Due to the lack of statistical significance associated with the existence of infection clusters, it was not possible to confirm an association between the occurrence of infection with one of these two agents and a specific geographic area within the study site. Nonetheless, a borderline significant cluster was detected in the central area of the city, compromising the municipalities of Santiago, San Miguel, and San Joaquin. Further analyses would be required to confirm the existence of this cluster and to determine the factors underlying its existence.

Monk Parakeets were introduced in Chile during the early 1970s, as fueled by the international pet trade market [17]. It is unknown if parakeets carried these pathogens upon their arrival or whether they acquired them in Chile. No reports of these agents existing within Monk Parakeets in their original distribution range have been issued, and although several endoparasitic studies have been performed on different Chilean birds [66,67,68,69,70], and more specifically on birds located in the city of Santiago [71], only *Cryptosporidium* spp. has so far been reported in Monk Parakeets [5]. As previously mentioned, nestlings most likely became infected via contact with adult individuals, which in turn may have acquired the pathogens from the surrounding environment. Infection with *Cryptosporidium* spp. and/or *Giardia* spp. is most commonly associated with the consumption of water contaminated with (oo)cysts [22,23,24]. Further studies should evaluate the quality of water sources present in public parks and squares. Interestingly, *Giardia* spp. was detected in soil samples from public parks in Spain [28], evidencing this substrate as a possible additional source of pathogens.

A different factor that could possibly be contributing to the presence of these parasites in Monk Parakeets relates to the coexistence of these birds and other domestic animals in public squares or parks. A study performed in 2012 detected the presence of *Cryptosporidium* spp. and *Giardia* spp. in fecal samples of dogs that were collected in public squares of the city of Santiago, Chile [72]. A similar study conducted in Spain also detected the presence of *Cryptosporidium* spp. and *Giardia* spp. in fecal samples collected in public parks of Madrid [28]. A second investigation, also in Madrid (Spain), detected the presence of *Giardia duodenalis* in dogs from an animal shelter [73]. This is a relevant finding since *G. duodenalis* has been previously described in aquatic birds [74] and has been isolated from a parrot [75]. Considering that in Santiago Monk Parakeets and dogs make use of the same environment to forage, and where Monk Parakeets also nest [20], the possibility of these agents being transmitted back and forth between parakeets and other species is possible. This also opens the possibility of Monk Parakeets being infected with other species of endoparasites and would also explain the general ubiquity of these protozoa in the studied area. Lastly, the possibility of Monk Parakeets contracting these pathogens from other bird species with which they share a common niche should also be considered, as it has been previously reported that Monk Parakeets in Santiago are able to peacefully forage and share space with other birds [20].

Interestingly, other species of birds make use of parakeet’s nests [20,21]. Such occurrence was recently registered in Santiago, Chile, where nine different species of birds, including Rock Doves and seven native species, were detected nesting inside Monk Parakeet’s nests [20]. Monk Parakeets often abandon their nest constructions in order to build new ones [76]. Additionally, incidents in which other birds attack Monk Parakeets, expel them from their nest, and later proceed to occupy it have been registered previously [77], and instances in which other bird species have been observed using empty chambers of parakeet nests that are otherwise still being occupied by parakeets have also been documented in Chile and other countries [6,20]. Regardless of whether Monk Parakeets abandon their nests voluntarily, are expelled from them, or share them, the sanitary implications of finding other birds inside these structures should not be overlooked. Coexistence in nesting sites, can eventually increase the transmission of parasites and diseases between different species [78]. *Cryptosporidium* spp. and *Giardia* spp. are orofecally transmitted parasites [23], and considering that Monk Parakeets defecate within their nests [12], it is quite possible that bird species using abandoned parakeet’s nests or making use of unoccupied chambers while parakeets still remain in the nest could eventually acquire these and other pathogenic agents, especially given that these protozoa may stay viable for weeks [79].

Monk Parakeets could also be spreading these parasites to other animals when being consumed by predators. American Kestrel (*Falco sparverius*) and Harris’s Hawk (*Parabuteo unicinctus*) have been reported as parakeet predators in Chile [20,80]. Recently, the role of Monk Parakeets as potential ecosystem engineers, providing a novel nesting resource in urban areas, was determined [20]. The present report shows that their role as ecosystem engineers could extend beyond the addition of a new nesting resources and include the potential role of Monk Parakeets as pathogens disseminators. This is supported by recent findings in Spain where Monk Parakeets were found to be infected with the beak and feather disease virus and the parasite Leucocytozoon [18,19].

As a synanthropic species, this may be of high health impact if some of these parasites are zoonotic, representing a major risk upon urbanized metropolises as Santiago, where these parakeets seem to be successful invaders.

Further, Monk Parakeet pathogen transmission risk is increased as this species tend to be favored by the public and are coveted as pets. In 2014, a domiciliary outbreak of psittacosis in the city of Dom Pedrito (Rio Grande do Sul State, Brazil) was attributed to Monk Parakeets after eight members of a family presented psittacosis-associated symptoms. Two parakeets illegally purchased by this family were identified as the source of *Chlamydophila* spp. [81]. Incidents such as this one could eventually increase and include the transmission of other pathogens.

For the time being, Monk Parakeets have remained confined to urban environments, but if they reach rural areas they could eventually encounter native Chilean parrots and transmit pathogens to them or to other bird species (e.g., beak and feather disease). This possibility is supported by a recent report issued in Spain that describes how Monk Parakeets from Madrid have spread into rural areas where they have been detected nesting in association with White Storks (*Ciconia ciconia*). This type of behavior could allow parakeets to increase their invasive potential by avoiding biotic resistance in the form of predator pressure, for example [82]. Although White Storks are not present in Chile, parakeets could eventually benefit from the presence of other bird species and expand their range of distribution.

Current environmental variations associated with climate change and agricultural land use can, generally, be positively associated with higher risks of contagion with *Cryptosporidium* spp. and *Giardia* spp. [83]. In addition to that, since infection can occur by consumption of contaminated water, control efforts for these pathogens usually focus on the sanitary management of water sources [84]. The present findings could contribute to highlight the importance of biological invasions in the emergence and control of diseases, especially those of urban densely populated areas.

It must be noted that despite exhibiting a wide variety of potential hosts, the *Cryptosporidium* and *Giardia* genus contain many different species and genotypes that differ in their ability to infect specific host species. This translates to a high level of species-specific infections. Since the identification of the parasites detected in this study was restricted to the genus identity of the pathogens, further analyses should be performed in order to determine the specific species infecting Monk Parakeets from the city of Santiago. Determining the species—and the genotypes—of the *Cryptosporidium* spp. and *Giardia* spp. (oo)cysts found in this study could help in assessing the real potential of these birds as possible transmitters of zoonotic agents to human and animal populations.

It should also be considered that even though the detection of *Cryptosporidium* spp. and *Giardia* spp. is important and the finding of pruned trees as a possible protective factor is interesting, further studies should be conducted in order to draw proper ecological and epidemiological conclusions about the presence of these pathogens and the environmental factors that influence their presence or absence. The sampling process carried out in this study was one of convenience, meaning that several arbitrary factors influenced the selection of the sampled trees. These ranged from nest accessibility to time and resources availability to permits granted by municipalities. For this reason, even though the statistical analyses performed in this work can reflect reality, they should still be considered with caution. Future studies on the sanitary state of Monk Parakeets should aim at a more thorough sampling design, ideally with a larger sample size or using a random sampling process not influenced by the previously mentioned factors.

## 5. Conclusions

This study corresponds with the second report of *Cryptosporidium* spp. in Chile and the first worldwide report of *Giardia* spp. in free-ranging parakeets from Santiago. Further studies focused on determining the specific identity of these protozoa, the presence of other pathogens in this invasive bird, and the existence of environmental variables favoring or impeding infections are of great importance, not only because of the potential transmission of pathogens to human beings, but also because, by coexisting with other bird species, this invader may by affecting the health of native birds in central Chile, one of the world’s 25 biodiversity hotspots and a priority for conservation measures [85].

## Figures and Tables

**Table 1:** Number of positive parakeets to *Cryptosporidium* spp. and to *Giardia* spp. per municipality.

Municipality	Total Number of Analyzed Samples	*Cryptosporidium* spp. Positive Samples	*Giardia* spp. Positive Samples
Conchalí	5	0	3
Huechuraba	4	0	0
Recoleta	20	1	2
Vitacura	8	0	2
Las Condes	22	1	1
Providencia	12	1	2
La Reina	37	2	2
Macul	5	0	0
San Bernardo	12	1	1
Peñalolen	13	2	1
Maipú	13	1	3
Puente Alto	5	0	0
La Granja	4	0	0
La Cisterna	1	0	0
Santiago	24	1	7
La Florida	3	0	0
San Miguel	9	0	0
La Pintana	5	0	1
Independencia	1	0	0
Pirque	3	0	0
Renca	1	0	0
TOTAL	207	10	25

**Table 2:** Results from the multivariable logistic regression analysis. Category, *p*-value, odds ratio (OR), and 95% confidence interval (CI) lower and upper limits are reported for the variables that were retained in the model showing an association or relevance on the positivity to *Cryptosporidium* spp. on Monk Parakeet samples.

Variable	Categories	*p*-Value	OR	95% CI	
				Lower Limit	Upper Limit
(Intercept)	-	0.001	-	-	-
Number of nestlings per nest	-	0.51	1.16	0.61	1.69
Nest height	-	0.82	1.00	0.99	1.01
Tree height	-	0.32	0.94	0.82	1.05
Tree canopy diameter	-	0.31	1.39	0.66	2.50
Pruning	-	0.06	0.14	0.01	0.83
Nest size	Big	Reference			
	Medium	0.24	0.17	0.009	4.99
	Small	0.19	0.14	0.008	4.11
	Mixed	0.21	0.14	0.006	4.47

**Table 3:** Results from the multivariate logistic regression analysis. Category, *p*-value, odds ratio (OR), and 95% confidence interval (CI) lower and upper limit are reported for the variables that were retained in the model showing an association or relevance on the positivity to *Giardia* spp. on Monk Parakeet samples.

Variable	*p*-Value	OR	95% CI	
			Lower Limit	Upper Limit
(Intercept)	<0.001	-	-	-
Number of nestlings per nest	0.003	1.59	1.17	2.17
Nest height	0.07	0.99	0.99	1.00
Pruning	0.01	0.25	0.09	0.71

**Table 4:** Final model results from the multivariate logistic regression analysis. Category, *p*-value, odds ratio, and 95% confidence interval (CI) lower and upper limit are reported for the variables that were retained in the model showing an association or relevance on the positivity to protozoan agents (*Cryptosporidium* spp. and *Giardia* spp.) on Monk Marakeet samples.

Variable	*p*-Value	OR	95% CI	
			Lower Limit	Upper Limit
(Intercept)	0.001	-	-	-
Number of nestlings per nest	0.39	1.21	0.80	1.87
Pruning	0.004	0.26	0.10	0.62
Age (days)	0.82	1.00	0.95	1.04
Nest height	0.21	1.00	0.99	1.00

**Table 5:** Number of positive samples, *p*-value, and 95% CI of prevalence differences to *Giardia* spp. and *Cryptosporidium* spp. between sampling seasons, obtained from introduced Monk Parakeets in Santiago, Chile.

Parasite	2017		2018		*p*-Value	95% Difference CI	
	Positive Samples	*n*	Positive Samples	*n*		Lower	Upper
*Giardia* spp.	10	98	15	109	0.568	−0.133	0.062
*Cryptosporidium* spp.	8		2		0.049	0.021	1.098

## Data Availability

The data presented in this study are openly available in FigShare at [https://doi.org/10.6084/m9.figshare.14204519], Table S1: Monk Parakeet Environmental Data.

## Supplementary Materials

The following are available online at https://www.mdpi.com/2076-2615/11/3/801/s1, Table S1: Monk Parakeet Environmental Data.
